# Epidemiology and control of human schistosomiasis in Tanzania

**DOI:** 10.1186/1756-3305-5-274

**Published:** 2012-11-28

**Authors:** Humphrey D Mazigo, Fred Nuwaha, Safari M Kinung’hi, Domenica Morona, Angela Pinot de Moira, Shona Wilson, Jorg Heukelbach, David W Dunne

**Affiliations:** 1 Department of Medical Parasitology and Entomology, School of Medicine, Catholic University of Health and Allied Sciences, P.O. Box 1464, Mwanza, Tanzania; 2 Department of Environmental Health and Communicable Disease Control, School of Public Health|, College of Health Sciences, Makerere University, P.O. Box 7072, Kampala, Uganda; 3 National Institute for Medical Research, Mwanza Research Centre, P.O. Box 1462, Mwanza, Tanzania; 4 Department of Pathology, Parasitology Section, Cambridge University, Tennis Court Road, Cambridge, CB2 1QP, UK; 5 Department of Community Health, School of Medicine, Federal University of Ceará, Ceará, Fortaleza, Brazil

**Keywords:** Schistosomiasis, *S. mansoni*, *S. Mansoni*, epidemiology, morbidity, control, Tanzania

## Abstract

In Tanzania, the first cases of schistosomiasis were reported in the early 19th century. Since then, various studies have reported prevalences of up to 100% in some areas. However, for many years, there have been no sustainable control programmes and systematic data from observational and control studies are very limited in the public domain. To cover that gap, the present article reviews the epidemiology, malacology, morbidity, and the milestones the country has made in efforts to control schistosomiasis and discusses future control approaches. The available evidence indicates that, both urinary and intestinal schistosomiasis are still highly endemic in Tanzania and cause significant morbidity.Mass drug administration using praziquantel, currently used as a key intervention measure, has not been successful in decreasing prevalence of infection. There is therefore an urgent need to revise the current approach for the successful control of the disease. Clearly, these need to be integrated control measures.

## Review

### Background

Human schistosomiasis is second only to malaria in sub-Saharan Africa (SSA) for causing severe morbidities. Of the world's 207 million estimated cases of schistosomiasis, 93% occur in SSA and the United Republic of Tanzania is the second country that has the highest burden of schistosomiasis in the region, Nigeria being the first [1,2]. Two major schistosome species are prevalent in the region, *Schistosoma mansoni* and *Schistosoma haematobium* causing intestinal and urogenital schistosomiasis, respectively [1,2]. In Tanzania, numerous surveys have been conducted in the past and more recently to describe the epidemiology, transmission (malacology), clinical trials of anti-schistosomes and control efforts against schistosomiasis (both intestinal and urogenital schistosomiasis), however, this information is very limited in the public domain where they can be easily accessed by public health intervention managers and policy makers. Availability of this information will not only help in implementation of control programs but also will serve to guide control activities in areas with the greatest needs and allocation of resources such as drugs. In attempts to cover that gap, the present article, reviews the epidemiology, transmission dynamics, past and current control approaches and the progress Tanzania has made to control the disease. Clinical features and treatments are discussed. Our review supports the need to reverse the current control approach based on preventive chemotherapy, to an integrated multidisciplinary control approach.

### Methods

Data for this review were identified and collected using manual and electronic search strategies of published and unpublished sources. Electronic databases included PubMed, EMBASE and Global health. References of relevant articles were also screened. Manual identification of unpublished literature sources, including unpublished project surveys, university theses, conference papers and public health organization reports was conducted at The National Institute for Medical Research, Mwanza, Tanzania. The centre has a library established in the 1940’s with archives of information since 1850.

To identify relevant studies on schistosomiasis in Tanzania, the search initially began with the text string “schistosom*” and a combination of the following words in permutations were used: “epidemiology”, “epidemiology + Tanzania/Tanganyika”, *“Schistosoma mansoni +* Tanzania/Tanganyika”, and “*Schistosoma haematobium +* Unguja *+* Pemba”, “schistosomiasis + Zanzibar”, “*Biomphalaria/Bulinus* + Tanzania/Tanganyika”. Available literature was categorized into population-based studies, clinical trials and basic malacology. Inclusion criteria were used with less stringency for the articles or papers that reported co-infections of schistosomiasis and other tropical diseases in Tanzania.

#### The past and current epidemiology of schistosome infections in Tanzania

Historical studies have revealed that both *Schistosoma haematobium* and *Schistosoma mansoni* have been endemic for a long time in Tanzania. The first scientific report of schistosomiasis in Tanzania can be traced back to 1895 when Manson-Bahr published the first recorded case of intestinal bilharziasis [3,4]. The early studies in the Lake Victoria province (Tanzania mainland) by Cook in 1905 at Kwimba identified and described the distribution of *S. mansoni* and *S. haematobium* in the region [3,5]. Over 50% of individuals examined had urogenital schistosomiasis [5] and *S. mansoni* was noted to be widespread on the southern and eastern shores of Lake Victoria [5]. In 1903, urogenital schistosomiasis was noted to be prevalent among men by Petrie in Zanzibar [6,7]. However, it was not until 1925 that urogenital schistosomiasis epidemiology was described [3,7]. Other schistosomes species found in the country include *Schistosoma bovis, Schistosoma matheei* and *Schistosoma leiperi* which infect bovine species [8,9].

Since the 1920s to date, large and small scale epidemiological surveys have been conducted in the country to determine the distribution [10-12], intermediate hosts [11-13], prevalences and intensity of both urogenital and intestinal schistosomiasis [14-18]. The results revealed that schistosomiasis was highly endemic throughout the country and the level of endemicity varied from region to region. The north-western regions, surrounding Lake Victoria, the northern, central, southern and south east of the country were identified to be highly endemic for *S. mansoni*[11,13,17],while the hinterland areas of the country were highly endemic for *S. haematobium*[14,15,18].

The distribution of *S. mansoni* and *S. haematobium* were reviewed by McCullough in 1972 and Doumenge in 1987 [3,19] and their reports indicated that the distribution of *S. mansoni* was found to be characteristically focal but the main extensive zones were found in the south eastern and south western sides of Lake Victoria and its island [19]. *Schistosoma haematobium* was widely distributed and two extensive zones where noted to have high transmission, namely the inland on the eastern and south-eastern hinterland of Lake Victoria and lowland zones on the eastern coast of the country [19]. The islands of Unguja and Pemba (Zanzibar islands) were only endemic to *Schistosoma haematobium* and the geographical distribution of the disease was largely restricted to north-western and central areas of Unguja Island [3,6,7,19-22]. However, Pemba Island, was endemic for *S. haematobium* on the western, southern, central and northwest of the island [23-27]. Two transmission seasons of the disease were noted in the islands part of the country: the high season, accompanying the long rains which end in between June/July, and low transmission during the dry seasons [28]. It was observed that *S. mansoni* was of little public health importance in the two islands due to absence of its intermediate hosts: snails, the *Biomphalaria* species [3,7,19].

The construction of the hydroelectric dams and development of irrigations schemes in the late 1970s and early 1980s to meet the needs of the growing population for food production and water supply altered the transmission pattern of schistosomiasis in the country. These water development projects were identified as the risk areas for transmission of schistosomiasis for communities that lived and worked in areas surrounding the hydroelectrical dams and irrigation schemes. The areas created favourable environmental conditions for the snail intermediate hosts and the major impact was that the disease was seen to spread to the areas that were not previously known to be endemic [12,29-35].

Of the national population of 17.5 million people (Tanzania mainland) in 1977, a total of 3.3 million (19%) people were conservatively estimated to have constant exposure risk to schistosomiasis [36]. According to the estimates in national surveys of 1979–1980, 28.3% of the population of mainland Tanzania (an approximate of 4.3 million people) and 23.2% (approximate 3.9 million people) had urogenital and intestinal schistosomaisis, respectively [36,37]. The nationwide survey conducted in 1980 among schoolchildren (aged between 9 and 14 years old) showed that more than 50% had *S. haematobium* infection, the majority living in the endemic zones of western and eastern parts of the country [3,36,37]. In 1990, for the projected population of 23.8 million people, the estimated national prevalence for schistosomiasis was 51.5% (an approximate of 12.3 million people with schistosomiasis infection) [36].

**Figure 1:**
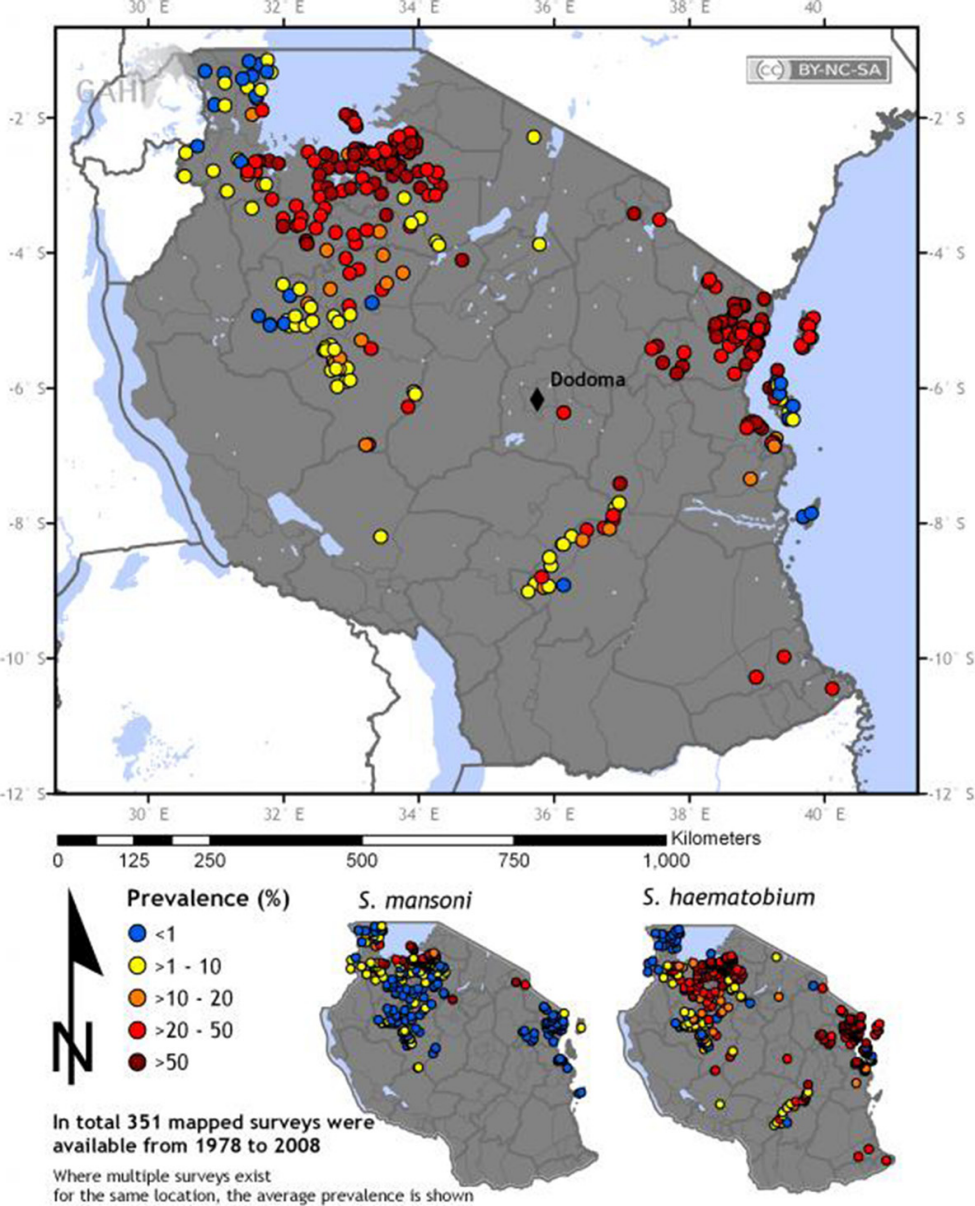
**Distribution of schistosomiasis (both *S. mansoni *** **and *S. haematobium*) in Tanzania: Maximum point prevalence of schistosomes infection and location of *S. mansoni *** **and *S. haematobium *** **surveys in the United Republic of Tanzania.**

Today, both urogenital and intestinal schistosomiasis remains as a major public health problem in Tanzania that are endemic at varying transmission levels in all administrative regions. *Schistosoma haematobium* is widely distributed while the endemic areas for *S. mansoni* are mostly focal [3,38,39]. Recently, attempts have been made to map the distribution and risk areas for schistosomiasis transmission using geographical information systems (GIS), remote-sensing (RS) and spatial analysis Figure 1[40-45]. The observed geographical distribution supports the previous historical mapping on schistosomiasis in the country. Specifically, *S. haematobium* is highly endemic along the eastern and south-eastern coasts, the islands of Unguja and Pemba (Zanzibar) and the interland areas of the north-western zones of the country [41]. These have been identified as potential areas for the intermediate-host snail species responsible for transmission of *S. haematobium*[41]. *Schistosoma mansoni* is absent on the coastal area due to the absence of its intermediate host snails and thermal exclusion [42,43] but is dominant along the shores and islands of Lake Victoria [19].

In the last decade, attempts have been made to estimate the population infected or at risk of contracting schistosomiasis in Tanzania [38,46]. In 2000, it was estimated that, of the 29.6 million people in Tanzania, 15.4 million were infected with both urogenital and intestinal schistosomiasis [37]. In 2009, it was estimated that approximately 19 million people are infected with schistosomiasis [47]. The recent report of 2012 indicated that of the estimated population of 43.5 million people, about 23,189,294 million are infected with schistosomiasis (the country prevalence estimated to be 51.5% [48]. Because of the wide distribution of schistosomiasis, the entire population of approximately 43.5 million people remains at risk of the disease and the prevalence of the disease appears to increase with increase in population size (from the prevalence of 19% in 1977 to approximately 51.5% in 2012).

#### Malacological survey and disease transmission

The early malacological surveys to incriminate fresh water snails as intermediate hosts of schistosomiasis started in 1879 in Unguja islands [46,48], where *Isidora* (*Physopsis*) *ovoidea* was identified as the likely intermediate host of *S. haematobium*[49,50]. Mozley in 1938 studied the molluscs of the continental part of the country, around the coastal area, the shores of Lake Victoria and the islands and identified *Physopsis globosa* as the intermediate host of *S. haematobium*[51].

The earlier reviews of Sturrock [12], McCullough [19] and Brown [51] in the 1970s and 1980s revealed that three Planorbidae were the principal intermediate hosts of *S. haematobium,* which included*Bulinus nasutus, Bulinus globosus* and *Bulinus africanus. Bulinus nasutus*occurred in the hinterland areas and was commonly observed in a variety of permanent water bodies, which were small and man-made, such as quarry excavations for road construction, rice paddies, swampy pools and temporary water sources throughout the country [3,12,51,52]. *Bulinusafricanus and B. globosus* were reported to inhabit permanent and larger water bodies both on the hinterland and coastal plain and were commonly seen in temporal streams [3,12,27,51,52]*.*

Molecular data and anatomical classification of the intermediate hosts of schistosomes have helped to resolve the taxonomic identification of important intermediate hosts of schistosomes in Tanzania [52-57]. Through the use of DNA characterization technique, it is now clear that on Unguja island *Bulinus globosus* is the main intermediate host of *Schistosoma haematobium*[53,56,58]. Although *Bulinus nasutus*has been identified to exist on the island, it is noteworthy that it is not the intermediate host of *S. haematobium*[53,56,58]. These observations indicate that the transmission of *S. haematobium* in Unguja could only take place within the distribution ranges of *B. globosus*[53,56,58,59]. On Unguja Island, the distribution of urogenital schistosomiasis corresponds with the distribution of *B. globosus*[53,56,58,59]. *Bulinus globosus*has been commonly observed towards the north of the island [56]. On Pemba Island, both *B. globosus* and *B. nasutus* are important intermediate hosts of *S. haematobium*, with the latter restricted to the eastern central border of the island but not widely spread [58].

In the mainland part of the country, there are several species of *Bulinus* and *Biomphalaria* responsible for transmission of urogenital and intestinal schistosomiasis [13,54]. The potential intermediate hosts of *S. mansoni* are *Biomphalaria choanomphala, Biomphalaria pfeifferi, Biomphalaria sudanica* and *Biomphalaria angulosa*[13,19]. *Biomphlaria choanomphala* is restricted to the large water bodies such as Lake Victoria and hydroelectrical dams in southern and east-south of the country, whereas *B. sudanica* is commonly observed in dams, in permanent and seasonal water sources around the southern shores of Lake Victoria and on the northern part of the country [12,13,19,56]. *Biomphlaria pfeifferi* has a widespread distribution and occurs throughout the inland (almost 2/3 of the country). The species is commonly observed in seasonal water sources and swamps whereas the *B. angulosa* is commonly seen in swamps in southern highlands [13,19,52]. For *S. haematobium*, the main intermediate hosts are *Bulinus africanus, B. globosus* and *B. nasutus*[19,60-62]. On the eastern coastal areas, *B. globosus* is the main snail host responsible for transmission [51]. However, *B. africanus* is also present in the area but has local importance [51]. By contrast, *B. nasutus* is the major intermediate host in north-western region and is commonly seen in temporary water bodies [15,19,54]. The geographical distribution of important snail intermediate hosts and attempted control measures are presented in Table 1.

**Table 1:** Malacological studies from different epidemiological settings and attempted control measures in Tanzania

**Genus**	**Host snail species**	**Geographical distributions**	**Potential hosts for**	**Attempted control measures**	**References**
*Bulinus*	*Bulinus globosus*	Unguja, Pemba, Pemba and all regions of mainland Tanzania.	*Schistosoma haematobium, -* is not intermediate hosts in Unguja	- On-going- Chemical control using (molluscicides-neclosamide) in Unguja and Pemba (http://www.score.uga.edu/Elimination.html www.controlled-trials.com/ISRCTN48837681/)	Sturrock., 1965 [12]
					McCullough [19]
					Webbe, 1966 [52]
					Stothard *et al*., [53]
					Lwambo,1988 [54]
					Mandahl-Barth [55]
				- Use of molluscicides/extracts from plants in mainland part of the country [63,64]	Stothard *et al*., [56]
					Stothard *et al*., [58]
					Webbe *et al*., 1958 [61]
					Zumstein, 1983 [62]
					Jordan and Webbe., 1982 [65]
					Stothard *et al.*, [66]
	*Bulinus nasutus*	Pemba, Mafia island and all regions of the mainland part of the country.	*S. haematobium, S. bovis*	On-going Chemical control using (molluscicides-neclosamide) in Unguja and Pemba (http://www.score.uga.edu/Elimination.html)	Mwambungu., 1988 [8]
					Kinoti., 1964 [9]
					Sturrock., 1965 [12]
					McCullough [19]
					Webbe, 1966 [52]
					Lwambo,1988 [54]
					Mandahl-Barth [55]
					Stothard *et al*., [56]
					Jelnes *et al*., [57]
					Marti *et al*., 1985 [67]
					Gabone *et al*., [68]
	*Bulinus africanus africanus*	Not present in Unguja and Pemba. Widely distributed on the mainland part of the country.	*S. haematobium, S. bovis, S. matheei, S. leiperi*	None	Doumange *et al*., 1987 [3]
					Mwambungu., 1988 [8]
					Kinoti., 1964 [9]
					Mutani *et al*., [69]
					Stothard *et al.*, [66]
	*Bulinus forskalii*	Probably all regions of mainland part of the country, no report from Unguja and Pemba. Present in the Mafia islands	*S. bovis*	None	Mwambungu., 1988 [8]
					Kinoti., 1964 [9]
					Mutani *et al*., [69]
*Biomphalaria*	*Biomphalaria choanomphala,*	- Confined in the large water bodies	*Schistosoma mansoni*	None	Magendantz., 1972 [13]
		– The lake Victoria. Common in the three regions bordering the lake.			McCullough *et al*., 1962 [19]
		- Absent on the eastern and southern coastal belt bordering Indian ocean, Unguja and Pemba.			Brown., 1980 [51]
	*Biomphalaria pfeifferi*	- All regions of mainland part of the country except the eastern coastal regions, Unguja and Pemba.	*Schistosoma mansoni*	- Chemical control (Bayer 73, N-tritylmorpholine in irrigations scheme northern Tanzania [63,70]	Utzinger and Tanner., 2000 [60]
					Webbe., 1964 [71]
				- Biological control [72,73]	Crossland., 1963 [70]
	*Biomphalaria sudanica*	All regions of mainland part of the country except the central regions of Dodoma, Singida, the eastern and southern coastal regions, Unguja and Pemba.	*Schistosoma mansoni*	- Molluscicides [74]	Doumange *et al*., 1987 [3]
					Magendantz., 1972 [13]
					Sturrock., 1962 [12]
	*Biomphalaria angulosa*	Common in lower lands and highlands southern	*Schistosoma mansoni*	None	Magendantz., 1972 [13]
					Sturrock., 1962 [12]
					Webbe and Jordan., 1966 [52]

The distribution and density of the intermediate snail host is an important determinant, accounting to a large extent for seasonal transmission of the disease [3,12,27,51]. The absence of members of the susceptible species of the planorbid genus *Biomphalaria* along the east coast areas and on the two islands of Unguja and Pemba is associated with the apparent absence of *S. mansoni* transmission in these areas [12,19]. Similarly, the distribution of *S. haematobium* and *S. mansoni* along Lake Victoria is largely related to the distribution of the intermediate hosts [65]. Along the shore of the lake members of the genus *Biomphalaria* are common [13,67] with populations living along the lake shores and islands being highly affected by *S. mansoni* as the risk of infection increases [67,68]. For *S. haematobium*, *Bulinus* species occurs far inland from the lake shores [14-16] and transmission of the infection is very common in the hinterland areas, except for parts of Kagera region where transmission does not occur due to the absence of these intermediate hosts [13,52,54,61,67].

#### Epidemiology and transmission

The prevalence, intensity of infection, and transmission intensity of schistosomiasis is determined by numerous factors including socio-economic, human behaviour, ecology and biological factors which influence the interactions between human and animal hosts and life cycle stages of the parasites.

#### Human water contact behaviours and transmission patterns

The various permanent and temporal water bodies existing in the country contribute significantly to the eco-epidemiological transmission of schistosomiasis. The inland water bodies on the southern shore of Lake Victoria contribute significantly to the transmission of *S. haematobium*[13,52,54,61,67]. Malacological and ecological surveys have identified several of the inland water bodies infested with *Bulinus* species (*B. nasutus, B. africanus* and *B. globosus*), which were shedding cercariae [61,68]. Large water bodies such as Lake Victoria, hydroelectrical dams and irrigations schemes (paddy and sugarcane irrigation schemes) are inhabited by *Biomphalaria* species (*B. chaonomphala, B.sudanica* and *B.pfeifferi*) [29-34]. The majority of the transmission of schistosomiasis occurs within the large water bodies, dams, irrigation schemes and in seasonal/temporal water bodies, predominantly around Lake Victoria shores and its islands. Dams and irrigation schemes for *S. mansoni* and the eastern coast belt and the Unguja and Pemba islands are predominant areas for *S. haematobium*[3,19]. To date, studies on human water contact and the influence of socio-economic factors on the transmission of schistosomiasis are very limited in Tanzania. These types of studies from other schistosomiasis endemic countries, using direct observations or interviews/surveys [75-78] have contributed significantly to identifying high risk individuals and groups in areas with focal transmission of the disease [28,75-78]. High water contact is a risk factor and a means of becoming infected for groups involved in fishing, farming, washing in water bodies and swimming [28,75-78]. Among these groups, high water contact behaviour is gender related. Males are reported to have higher water contact behaviour than females [28,75-78]. In addition, high water contact in males has been observed to correspond with a high peak of cercarial shedding [28,75-78]. Age-related water contact behaviour has also been observed. Studies in Africa have reported high water contact in the age group < 21 years and other studies in age group > 21 year [28,75-78].

**Figure 2:**
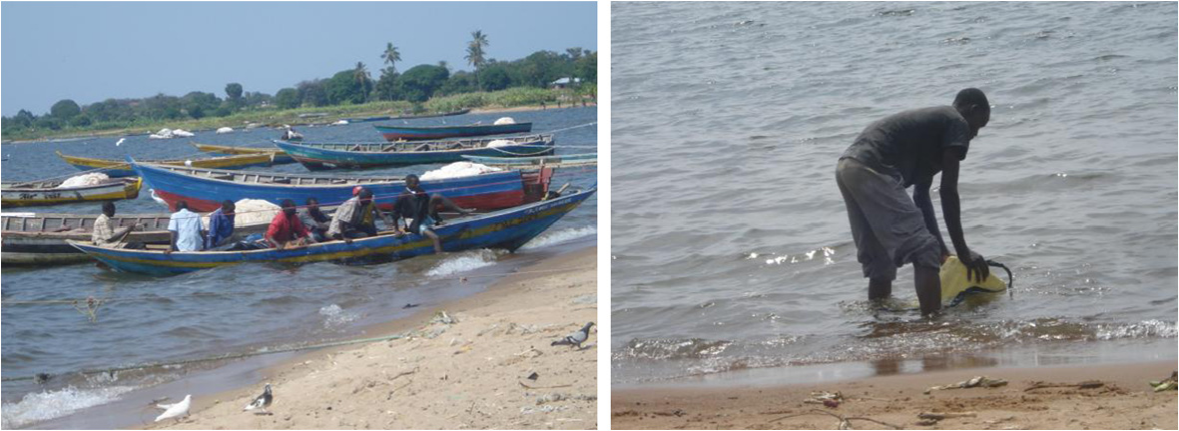
**Active water contact activities such as fishing, fetching water direct from the lake and agriculture along the lake basin increases the risk of *S. mansoni *** **transmission on the southern shore of the Lake Victoria, north-western Tanzania.**

In Tanzania, human exposure to schistosomiasis is mostly related to occupational activities such as fishing, farming or recreational activities around the basin or within the permanent or temporal water bodies such as lakes, rivers, dams, swampy areas or road side ditches. There is variation in water contact behaviour from one region to another region due to differences in distance to water bodies, socio-economic conditions and ecological variation [28,75-78] Figure 2.

The transmission pattern of schistosomiasis is seasonal and alternates between rainy and dry seasons [15,16]. In areas surrounding the southern shore of Lake Victoria and on Unguja Island, two distinct transmission seasons of *S. haematobium* have been observed [13,16,52]. The first season is observed between February- May and is concurrent with the long rainy seasons [13,16,52]. During this season, the number of temporal water bodies in hinterland increases, volume of water in seasonal rivers increases and the female *Bulinus* species starts to lay a large number of eggs which hatch by the end of long rainy season [15,16]. Thus, shortly after the long rainy season, the population density of *Bulinus* increases and both young and mature snails shed cercariae at high rates [13,16,52]. At the start of the dry season, between June and July, the ambient temperature increases and creates a favourable environment for human water contact through swimming and fishing. This exposure to cercariae infested water increases the rate of disease transmission [13,16,52]. The second transmission season is observed during the dry season (July – September/October) characterized by decreased *Bulinus* population density, drying out of most of the temporal water bodies, decreased water volume in seasonal rivers and no agriculture work in the hinterland areas [13,16,52]. The adult snails die during the dry season and the young snails undergo aestivation under the soil to ensure survival during the next season and species survival adaptation [13,16,52]. During this period, the transmission rate is low and sporadic. However, in other areas the transmission pattern is observed shortly after the short dry season [54].

For *S. mansoni*, in the hinterland areas, the transmission pattern follows that of *S. haematobium* due to seasonality of water bodies. However, in permanent water bodies, such as around Lake Victoria, irrigation schemes or around hydroelectrical dams, the transmission is different and does not occur in seasons. In these areas, there is continual transmission with slight or no difference between the rainy and dry season [13,15,16,52].

#### Primate - Human schistosomiasis transmission

The zoonotic transmission of *S. mansoni* between human and other primates occurs in Tanzania, but this topic has received little attention [79-81]. Monkeys and baboons are known to be infected by *S. mansoni* in their ecological areas [79-81]. Studies involving these primates in their vicinity or protected national parks have reported high incidence of *S. mansoni* in monkeys and baboons. These primates act as reservoirs for the infection to humans and play a major role in the transmission of the disease [79-81]. However, the potential roles played by monkeys/baboons as reservoir hosts of human schistosome infections have never been studied in detail. The movement of baboons from the national parks to human settlements may establish transmission of intestinal schistosomiasis in areas known to be free of the disease, especially if the intermediate hosts are present in that area. Thus, advanced studies are required to understand the roles played by baboons/monkeys in the dynamic transmission of intestinal schistosomiasis.

#### Population prevalence and intensity of infection

Like other schistosomiasis endemic countries, in Tanzania the prevalence of schistosomiasis and intensity of infection varies between geographical areas, age groups and gender. Studies have reported high prevalences (40% to 100%) of *S. mansoni* in populations living along the Lake Victoria shores and within islands, irrigation schemes and around the hydroelectrical dams [5,17,44,45,82-84]. For *S. haematobium*, the high prevalence is observed in hinterland areas located on the southern shore of Lake Victoria, the eastern coastal areas and on Unguja and Pemba islands [3,10,11,14,15,18,19]. Prevalence and infection intensity are highest among school children <15 years of age [36,37]. The age distribution pattern of schistosomiasis has been reported to peak between ages 10–19 years and decrease there after with increased age [36,37]. Similarly, a gender variation in prevalence of *S. mansoni* appears to have a variation and the variations are attributed to differences in economic activities and exposure to cercariae infested water. Males are exposed more to water during fishing and swimming as compared to females [36,37,85]. In terms of age variation in prevalence, community studies have observed individuals aged < 30 years having higher *S. mansoni* prevalence as compared to those who were > 30 years [83-85]. Similarly, for *S. haematobium* infection an age-dependent pattern of infection peaks at the age group of 5–19 years old and a decrease in the >20 year-olds [86]. The intensity of infection differs significantly between males and females with mainly young age groups (14–19 years old) diagnosed with heavy infection both for *S. mansoni* and *S. haematobium*[5,82,83,86]. Development of immunity against schistosomiasis is attributed to the age pattern observed and the decrease of the prevalence and intensity of infection at older ages.

In communities where occupational exposure is unavoidable for a large proportion of the adult population, for example in fishing and farming communities, adults are more exposed to cercarial infested water than the young generation due to their occupations, such as paddy cultivation in swamps or fishing in lakes [54,65,86]. This age difference in prevalence and infection intensity can also be influenced by school based control approaches, in which the control intervention is only centered around school children and the adult population is not included, which has been for example, observed in China and the Philippines [87,88].

#### Schistosomiasis pathology and patterns of morbidity

Chronic schistosomiasis (both intestinal and urogenital) results from the host’s immune response against the egg antigens (hepatotoxins -Omega-1) which reach the body tissue through the micropores in the egg shells and [89,90]. The hepatotoxins provoke an eosinophilic response and type IV (delayed) hypersensitivity reactions mediated through T helper cell (Th_2_) immune response to antigens [89,90]. The immunological reaction produced eventually leads to formation of granuloma, which ends up as a fibrosis formation [89,90]. The resulting pathology depends on several factors such as host-parasite genetics [91], degree and length of exposure [92], intensity of infection [92], host immune response to the parasites and co-infections with other tropical diseases such as malaria and HIV-1 [93,94]. Chronic schistosomiasis associated with immune response against eggs trapped in various body tissues is commonly reported in Tanzania [4,83,84,86].

#### Chronic urogenital schistosomiasis

*Schistosoma haematobium* worms are strategically located at the terminal venules, which tend to enhance passage of the laid eggs to the lumen of the bladder, pelvic organs and rarely in the gastrointestinal organs [95]. The deposited eggs provoke granulomatous inflammation and the primary organ affected is the ureter [89]. The developed lesions lead to the formation of sandy patches, ulcerations and polypoid lesions in the bladder and ureters, resulting in haematuria, obstructions of urine flow, calcified bladder wall and cancer [14,18,95,96]. Common early signs include dysuria, proteinuria, and haematuria [95-111]. The development of urological abnormalities related to *S. haematobium* infection is associated with the intensity of infection [96,112-115]. Several epidemiological and clinical studies conducted in Zanzibar (Unguja and Pemba) and the mainland part of the country have reported high prevalences of *S. haematobium* and different types of morbidities associated with urogenital schistosomiasis across all age groups and gender (Table 2). The common urogram abnormalities were calcified urinary bladder, calcified and distorted ureters, hydronephrosis and non-functioning kidney [96,112-116]. In addition, multiple or bilateral lesions were also identified, including calcified bladder, deformity of the ureter, hydronephrosis and non-functioning kidneys [112-116]. A direct relationship between intensity of infection and the abnormalities of the urinary tract was observed, in which calcified urinary bladders, deformed ureters and hydronephrosis were significantly more common in schoolchildren who had higher infection intensities (excreted >250 eggs/10ml of urine) [112-115]. Similar findings were reported in Unguja and the observed urological complications were associated with high intensity of infection [112-115]. Ultrasonographical surveys in the south east of the country, the areas endemic for *S. haematobium,* produced similar results, but the overall rate of urological abnormalities was high [96]. Recently, factors such as pain on micturating, urgency, loin pain, tired by midday, self-reported frothy urine, self-perceived haematuria, self-reported schistosomaisis in past year, detected haematuria and presence of *S. haematobium* eggs in urine were major risk factors for urinary tract abnormalities detected by ultrasound [116]. Details of the results of other studies on morbidities associated with urogenital schistosomiasis are presented in Table 2.

**Table 2:** Epidemiological and clinical studies reporting prevalence, intensity of infection and morbidities associated with schistosomiasis in Tanzania

**Articles**	**Parasites**	**Major findings**	**Geographical area**
Jordan, 1961 [14]	*S. haematobium and S. mansoni*	A total of 203 individuals were examined. Higher incidence of *S. haematobium* in the age group 6–12 year olds (78%) and 50% in the > 12 years of age. Only 12 individuals had *S. mansoni* ova in their stool.	Usagara, Sukuma-land, north-western Tanzania
Forsyth and Bradley, 1966 [113]	*S. haematobium and S. haematobium*	A total of 2,338 people (1580 males, 758 females) were studied. Overall prevalence was 42% (42% *vs* 41% between males and females) and the age group 6–17 years having a prevalence of 69%. The prevalence of *S. mansoni* was 24% with the majority having light infection and showing a sex difference in prevalence, being common in men (31% versus 15%). *Schistosoma mansoni* infection was common in individuals living in villages close to the lake and *S. haematobium* was higher in villages located in the hinterland.	Bukumbi chiefdom, north-western Tanzania.
		- Prevalence of splenomegaly (Hackett grade 2 or more) was 25% and 8% for individuals identified to have *S. mansoni* infections.	
		- Splenomegaly and hepatomegaly was also seen in individuals who had no *S. mansoni* infections and was presumed to be caused by malaria infection.	
Forsyth and MacDonald 1965 [112]	*S. haematobium*	Three urine samples collected from each of the 517 school children. The overall prevalence of *S. haematobium* was 45.3% (234/217) and sex difference in prevalence was observed with boys having a prevalence of 51% versus 37% in girls. Infected children were observed to excrete up to more than 4000 eggs/10mls of urine. The prevalence of *S. haematobium* was observed to vary with villages divided as “good and bad villages”. In terms of pathological lesions, 43.4% had different urological lesions (calcified bladder, deformed ureters, hydronephrosis) from “bad villages” and 17.4% from “good” villages.	Unguja (Zanzibar)
McMahon 1967 [82]	*S. mansoni and S. haematobium*	640 individuals examined and 200 went through a thorough clinical examination. The prevalence of *S. mansoni* was 65.2%, 12.2% for *S. haematobium* and 3.4% had mixed infections (*S. mansoni* and *S. haematobium*). On clinical examination, 57% and 19.3% of individuals of different age groups infected with S. mansoni had palpable splenomegaly and hepatomegaly. Similarly, 29.4% and 12.8% of the individuals infected with only *S. haematobium* had palpable spleens and liver. Other parasitic infections such as malaria contributed to hepatomegaly and splenomegaly.	Mwanza and Ukerewe, north-western Tanzania.
Forsyth, 1969 [114]	*S. haematobium*	A two year longitudinal study which studied 1004 people. Overall prevalence of *S. haematobium* was 65.1% and the prevalence was higher in the age group between 7 and 17 years (90% - 100%). The prevalence of urological abnormalities (based on urograms results) was 35.4%.	Unguja (Zanzibar)
		- The infection intensities (number of eggs excreted) were reported to exceed 2000 eggs/10mls of midday urine.	
		- Haematuria was common in young children and less in older and adult individuals. The urograms of 201 (45.8%) males and 80 (22.5%) were abnormal. The overall prevalence of pathological lesions was 35.4% [calcified bladder 14%, deformed ureters 23.8%, hydronephrosis 15%, non-functioning kidney 4.5% and stones 0.5%]. These pathological lesions were more common in males than in females and were observed to increase with age.	
Rugemalila., 1981 [18]	*S. haematobium*	900 individuals examined from two communities, prevalences of *S. haematobium* ranged from 54% to 57% with peaks of 66% - 67% among the 5–9 years olds. Almost 90% of the infected individuals had symptomatic vesical lesions and radiological examination of 100 individuals selected randomly revealed that 28% had bilateral uretero-renal lesions.	Mwanza, north-western Tanzania.
Zumstein, 1983 [98]	*S. haematobium*	3,478 school children examined for *S. haematobium*. The overall prevalence of *S. haematobium* infection among the school children (6–19 years) was 21%. The highest prevalence was observed in both sexes in the age group 15–19 years. Distinct variations in prevalence were found between the individual schools examined, ranging from 5 to 71% and indicating a focal transmission of the disease. The intensity of *S. haematobium* infection in the individual schools was relatively low, ranging from 5 to 36 eggs/10 ml urine. However, the frequency of microhaematuria among infected subjects was high, reaching 100% from an egg output of 50 eggs/10 ml onwards. Forty-nine water-bodies, most of them man-made-with *Bulinus* (Ph.) *globosus* and/or *B.* (Ph.) *nasutus* were identified.	Ifakara, South-Eastern Tanzania.
Sarda *et al*., 1985 [99]	*S. haematobium*	2,500 school children from 12 primary schools. The prevalence in the schools ranged from 5.3 to 55.1%, with an overall prevalence of 19.3%. More males (23.5%) than females (15.0%) were infected, and the highest prevalence was recorded in the 11–16 year old age group. Intensity of infection was higher, ranging from 12 to 96 eggs/10 ml urine in individual schools. 26% of the infected excreted more than 50 eggs/10 ml urine and high rates of haematuria and proteinuria were observed in infected children. Malacological surveys showed two potential vectors, *Bulinus globosus* and *B. nasutus*.	Dar Es Salaam, Eastern Coast, Tanzania
Kitinya *et al*., 1986 [117]	*S. haematobium*	Histopathological examination of 172 cases of urinary bladder cancer, 72% had squamous cell carcinomas and 46% had *S. haematobium* eggs.	Northern Tanzania
Savioli *et al*., 1990 [25].		520 individuals examined for visual haematuria and parasitological examination. Strong variability of day to day of egg excretion within the study participants was observed both in the whole population and the age group 5–19 years. The prevalence of participants excreting one or ≥ 50 eggs/10 ml of urine ranged between 36% - 61%. Gross haematuria had higher specificity (100%) in relation to positive filtration on any day of examination and egg counts of ≥ 50 eggs/10mls of urine. Also a positive reaction of haematuria of any day of examination was associated with the study participants having a high egg count (≥ 50 eggs/10ml urine).	Pemba
Savioli *et al.* 1989 [26]	*S. haematobium*	879 individuals examined for *S. haematobium* and 520 had six days complete urine samples examination. Overall prevalence based on urine filtration technique was 26-34% and in the age group 5–19 years and the highest prevalence of heavy infection was in the age group 10–14 years. The prevalence of haematuria detected by the reagent strips was similar to prevalence of infection as measured by the highest egg counts. Thus, urine reagent strips are useful diagnostic indicators of *S. haematobium*.	Pemba Island
Mgeni *et al*., 1990 [101]	*S. haematobium*	4,113 individuals examined at least once during two years period. First survey, 2,685 individuals examined, 49.3% had *S. haematobium* infections and 19.3% with heavy infection. The age group 10–14 years had higher prevalence of heavy infection (30.2%). Second survey, 1887 examined, 39.8% had infection and 13.5% with heavy infection. The age group 5–9 years old, 27.2% had heavy infection. Third survey, 2,458 individuals were examined, 34.5% had infection and 10.9% were heavily infected. Fourth survey, 1719 individuals were examined, 23.2% had infection and 7.3% were heavily infected. *S. haematobium* is highly endemic in the areas and the age group 5–14 years had highest prevalence and heavily infected.	Zanzibar
Albonico *et al*., 1997 [107]	*S. haematobium*	3,605 school children examined for *S. haematobium* and other parasitic infections, 31% of the children had detectable haematuria and about 67% of them had co-infections of *S. haematobium* and geohelminths.	Pemba Islands
Kardorff *et al*., 1997 [83]	*S. mansoni*	Parasitological and ultrasonographical examination of 1,659 and 898 individuals for *S. mansoni*. High prevalence and intensity of infection in all the three villages was observed, with children and adolescents more infected than adults, and males excreting more eggs than females. Overall prevalence of *S. mansoni* infection was 86.3% and mean egg output was 514 epg (geometrical mean egg output was 161.1 epg). Prevalence of hepatomegaly (detected by ultrasonography) was 35% and 80% had splenomegaly. Organomegaly was strongly associated with heavy epg. For periportal fibrosis, 29.8% had grade I, 5.1% had grade II, 1.1% had grade III and fibrosis was observed to increase with age. Late stage disease with a high degree of periportal fibrosis, shrinking of the right lobe of the liver and features of portal hypertension was seen in 2.1% of the individuals. The overall level of schistosomal morbidity was classified as intermediate.	Ukerewe, north-western Tanzania.
Hatz *et al*., 1998 [96]	*S. haematobium*	533 school children examined for urinary tract pathologies. Baseline data collection found urinary tract pathology in 67% of 533 children. Lesions of the bladder were significantly associated with egg positivity and microhematuria. The attributable fraction estimate of major bladder lesions due to *S. haematobium* was 75%. In a cohort study, 224 infected children were examined by ultrasound and then treated with a standard dose of 40 mg of praziquantel/kg of body weight. They were re-examined at two, four, six, 12, 18, and 24 months after treatment. Before treatment, 76% had pathologic lesions of the urinary tract. The proportion showing lesions decreased sharply during the first months after treatment to 11% at six months. At 24 months, lesions were detected in 57%, and 11% had developed new severe pathology. In 18 cases, pathology was present throughout, and 34 did not show any pathology throughout the study.	Ifakara district, southeastern Tanzania
Lwambo *et al*., 1999 [110]	*S. haematobium, S. mansoni* and geohelminths	6,897 school children aged 7–20 years. Parasitological examination of *S. haematobium* and *S. mansoni*. Prevalence of *S. haematobium* was 56.5%, *S. mansoni* 10.9% (overall schistosomiasis infection was 63.4%) and mixed infection at 4% (*S. haematobium* and *S. mansoni*). *Schistosoma haematobium* infections was observed to increase with age.	Magu district, north-western Tanzania.
Ndyomugyenyi and Minjas 2001 [97].	*S. haematobium*	1,200 schoolchildren examined. The overall prevalence, based on microscopic examination of a single urine sample/subject, was 47.6%. Compared with the girls, the boys were more likely to be excreting schistosome eggs (54.6% versus 40.8%; *P* = 0.004) and they had higher intensities of infection (54 versus 38 eggs/10 ml urine; *P* = 0.001). The children aged 10–14 years had higher prevalences and intensities of infection than those in the younger or older age-group studied.	Dar Es Salaam, East Coast, Tanzania
Stothard *et al*., 2002 [106].	*S. haematobium*	400 school children from ten different primary schools were examined for *S. haematobium*; the overall prevalence was 12% with five schools recording no infection. Primary schools on the west of 39^o^ degrees 19'E and north of 6^o^ 10'S harboured nearly all the infections, with highest prevalence (55%) recorded in an area with a high number of *B. globosus* habitats. The knowledge of schistosomiasis was poor and individuals self-diagnosis was poor (sensitivity, 8.5%). Freshwater-contact patterns of schoolchildren differed significantly between schools and correlated well with prevalence of infections within schools.	Unguja (Zanzibar)
Poggensee *et al*., 2005 [109]	*S. haematobium* and *S. mansoni*	634 school children examined in two villages, prevalence of *S. haematobium* was 37% and 86.3% respectively. For *S. mansoni*, prevalence was22.9% and 43.5% among the school children. Six years after the intervention, the prevalence of S. haematobium remained almost constant, at 33.5% and 70% in the two villages. The prevalence of heavy infection (≥ 50 eggs/10ml urine) decreased from 53.8% to 34.4%.	Mwanga district, northern Tanzania
Rollinson *et al*., 2005 [108]	*S. haematobium*	305 schoolchildren examined. Prevalence of *S. haematobium* based on parasitological examination was 53.9%. Negative association between haemoglobin level and *S. haematobium* infection intensity was observed. An association between reported pain during micturitions and elevated urine-albumin levels was observed.	Unguja (Zanzibar)
Ajanga *et al.* 2006 [118].	*S. mansoni*	972 pregnant women examined. Overall, 63.5% were infected with *S. mansoni* with prevalence being highest among younger women and decreased with increased age. Overall, 66.4% of the women were anaemic and the increased risk of anaemia was associated with heavy (≥ 400 epg) infection of *S. mansoni*.	Ukerewe, north-western Tanzania
Rudge *et al*., 2008 [59].		150 school children examined for *S. haematobium* and a questionnaire to assess water contact pattern. Overall prevalence of *S. haematobium* was 50.6% with more boys frequently and more heavily infected than girls. In addition, the mean exposure scores was significantly higher in boys than girls. Water contact activities and proximity of children’s home to a site harbouring *S. haematobium*-infected *B. globosus* were associated with *S. haematobium* infection.	Unguja (Zanzibar)
Malenganisho *et al.* 2008 [119].	*S. mansoni*	Parasitological and ultrasonographic examination of 1,447 individuals from two communities (aged 14–87 years). The prevalence of *S. mansoni* was 78% and 38%. The geometrical mean egg output was 156 epg and 47 epg for the two communities respectively. The prevalence of periportal fibrosis was 41.5% and 16.7% and was associated with high prevalence and intensity of *S. mansoni*. Periportal fibrosis, increased segmental branch wall thickness and dilated portal vein diameter were more common in males than females.	Ukerewe and Ilemela district, north-western Tanzania.
Lyons *et al*., 2009 [116]	*S. haematobium*	160 from north (highly endemic) and south (low endemic) of the individuals screened for *S. haematobium* and ultrasonographical examination of urinary tract morbidities. Individuals from endemic areas had higher urinary tract morbidities as compared to individuals from a low endemic area. Having two out of urgency urination, self reporting of previous infections and detection of eggs in urine were good proxy predictors of urinary tract morbidities as detected by ultrasound.	Unguja
Sousa-Figueiredo *et al*., 2009 [104]	*S. haematobium*	147 school children and 47 adult men examined for *S. haematobium* prevalence and urinary tract pathologies. The prevalence of egg-patent urinary schistosomiasis was 36.4% and 46.8% and that of urinary tract pathologies was 39.4% and 64.4% respectively. In school-children, raised urine-albumin concentration (>40 mg/L) was not associated with the prevalence of *S. haematobium* but was strongly associated with the prevalence of micro-haematuria (76.7, *P*<0.0001). In adults, elevated urine-albumin excretion was associated with urinary tract pathologies, particularly lesions of the bladder was (OR=8.4, *P*= 0.013). Albuminuria was a good indicator of detecting lower urinary tract pathologies (bladder wall lesions).	Unguja
Stothard *et al*., 2009 [105]	*S. haematobium*	66 children examined for urogenital schistosomiasis and urinary tract pathologies. Prevalence of egg-patent schistosomiasis was 65.2%, while 77.3% had micro-haematuria and 66.1% had at least one ultrasound-identified urinary tract pathology.	Unguja
Massa *et al*., 2009 [111].	*S. haematobium* and *S. mansoni*	585 children from the community and 555 children from school were examined for *S. haematobium, S. mansoni* and geohelminths. The prevalence of *S. mansoni* was 29.4% for children from the community and 26.8% for school children. For *S. haematobium* was 27.6% and 25.2% respectively.	Umba division, Lushoto district, Tanga. Northern Tanzania.
Poggensee *et al*., 2000 [120]	*S. haematobium*	657 women examined for *S. haematobium* and the prevalence was 36% and median intensity of infection was 2.1eggs/10ml of urine. The proportion of schistosomiasis of the lower urinary tract on gynecological examination among 359 women was 37% and the proportion of their urinary schistosomiasis was 42%. Among the 134 women with Female Genital schistosomiasis, 56% had detectable eggs in urine. Cervical lesion occurred in 75% of the women with female genital schistosomiasis.	Mwanga district, Kilimanjaro region, Northern Tanzania.
Downs *et al*., 2011 [121]	*S. haematobium and S. mansoni*	457 women aged 18–50 years were examined for female urogenital schistosomiasis. The prevalence of female genital schistosomiasis was 5% (ranged from 0% - 11%) and female urogenital schistosomiasis was associated with HIV infection and younger age. Overall HIV prevalence was 5.9% but was 17% among women with female urogenital schistosomiasis. A significant geographical clustering of schistosomiasis was observed: northern villages near Lake Victoria had more *S. mansoni* infections, and southern villages further from the lake had more *S. haematobium*.	Sengerema and Misungwi districts, north-western Tanzania
Scheich *et al*., 2012 [122]	*S. mansoni*	360 schoolchildren (aged 6–17 years) were parasitologically and ultrasonographically examined, 62 % and 57.7% of males and female were infected with *S. mansoni* respectively. The infection intensity ranged from 1–2,440 epg with 32% having low grade, 53.2% having moderate and 14.8% having heavy infection intensities. On ultrasonographical examination, 90.7% had splenomegaly, 89.3% and 30.9% had right and left lobe hepatomegaly. Only 5.4% of the children had overt signs of portal fibrosis and 28.5% had portal vein dilatation. No association was observed between portal fibrosis and infection intensities.	Ukerewe district, north-western Tanzania.
Stothard *et al*., 2012 [66]		238 children from nine primary schools were examined for *S. haematobium*. The prevalence of micro-haematuria and egg-patent infection was 18.1% and 4.2% respectively. Females had higher prevalence of micro-haematuria compared to males. All egg-patent infections were of light-intensity (<10 eggs/10ml). No clear associations between infection prevalence and local water-contact, by school, were found and all 10 of the egg-positive children had a travel history to the nearby mainland or Zanzibar. Retrospective hospital data revealed a low proportion (< 2%) of egg-patent infections for 20,306 samples tested between the 2000–2005. Malacological survey revealed that four *Bulinus* species were common in the area (*Bulinus nasutus, B. forskalii, B. barthi*). No collected snail was observed to shed schistosome cercariae.	Mafia district, Coastal region, Tanzania.

**Figure 3:**
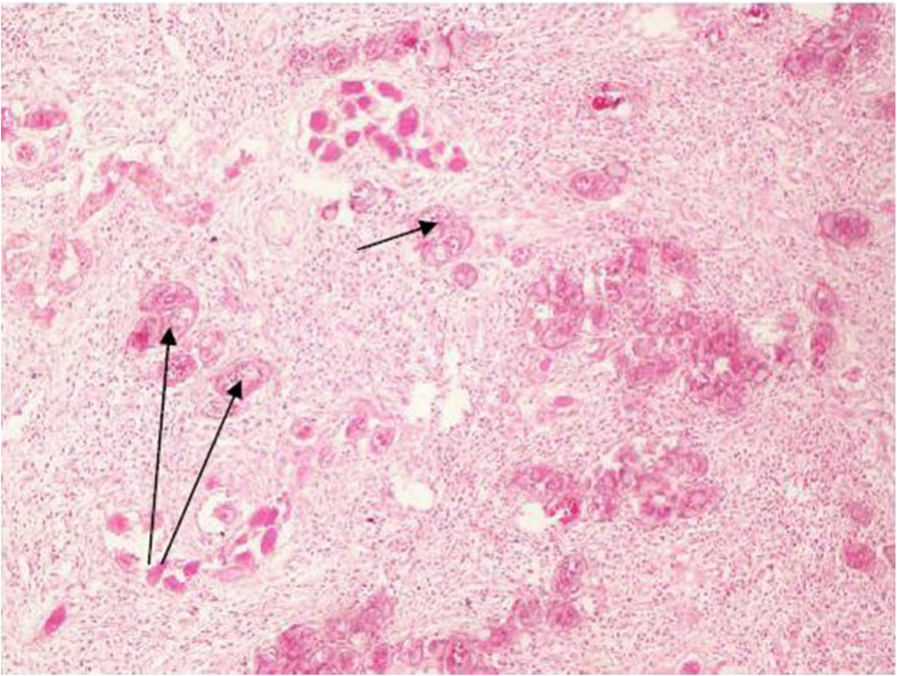
**Arrows showing eggs of *S. mansoni *** **(with lateral spine) in the submucosa and mucosa of the ileum and caecum of a 7 year old Tanzania boy presenting with intestinal.**

In Tanzania, genital schistosomiasis due to eggs of *S. haematobium* trapped especially in the female reproductive tract is a public health concern [120,123-125]. Symptoms such as bleeding disorders, ulcers, lower abdominal pain and infertility are common among infected women [120,123-125]. Gynecological and pathological findings are mainly confined to the cervix, where common findings include so-called sandy patches, oedema, erosions/ulcerations, and polyps [120,123-125]. In addition, co-occurrence of genital *S. haematobium* infections and carcinoma of the uterine cervix or carcinomas of the urinary bladder or prostate gland have been reported Figure 3[117,125,126]. The association between *S. haematobium* infection and squamous-cell carcinoma of the bladder has been the subject of debate for a long time. Co-existence of squamous-cell or adenocarcinoma of the urinary bladder, prostate gland and eggs of *S. haematobium* has been observed in hospitalized cases [117,125,126]. Evidence from other studies indicates an association between *S. haematobium* infections and some types of bladder cancers [95]. In addition, *S. haematobium* associated lesions in the reproductive tract of women may facilitate the transmission of other sexual diseases including HIV (Table 2) [121,127].

#### Chronic intestinal schistosomiasis

The predilection sites of *S. mansoni* are the mesenteric veins. Eggs from the fertilized adult female worm tend to transverse the large bowel walls, causing mild to severe gut pathologies [128,129]. In mild infections, the symptoms include diarrhea, abdominal pain, bloody stool, nausea, fatigue and drowsiness [83,129]. Eggs which do not manage to pass through the intestinal mucosa to reach the lumen are trapped in the mucosa or other parts, and are swept by the portal blood flow. Because the eggs are too large to pass through the sinusoidal plexus, they accumulate in the presinusoidal venules within the portal triads [128,129]. Immunological response against the accumulated eggs results in extensive fibrosis of the liver, which may lead to hepatomegaly or splenomegaly/hepatosplenomegaly, venous obstructions, portal hypertension and development of oesophageal varices [128-130]. The rupture of collateral veins in oesophageal varices results in haematemesis [129].

Detailed liver pathology, liver function and haematological findings associated with *S. mansoni* identified and reported by various epidemiological and clinical surveys are presented in Table 2[17,82-84,119,122]. In clinical studies common symptoms reported include vague abdominal pain, general weakness, diarrhea, intermittent dysentery and blood/mucus in stool [82,83,119]. Ultrasonographical examination has revealed high prevalences of hepatomegaly and hepatomegaly, splenomegaly, hepato-splenomegaly and portal fibrosis. A consistent increase in the proportion of hepatomegaly and splenomegaly with increasing egg output has been observed. Individuals with heavy infections had higher prevalence of both hepatomegaly and splenomegaly [83,84,119,122]. Similarly, histological findings have demonstrated extensive fibrosis and granulomatous lesions in liver sections of individuals who had heavy infection intensity (≥ 500 epg) [82]. In addition, portal tract fibrosis was severe in liver sections with ≥ 250 eggs of *S. mansoni* per gram of faeces [82].

**Figure 4:**
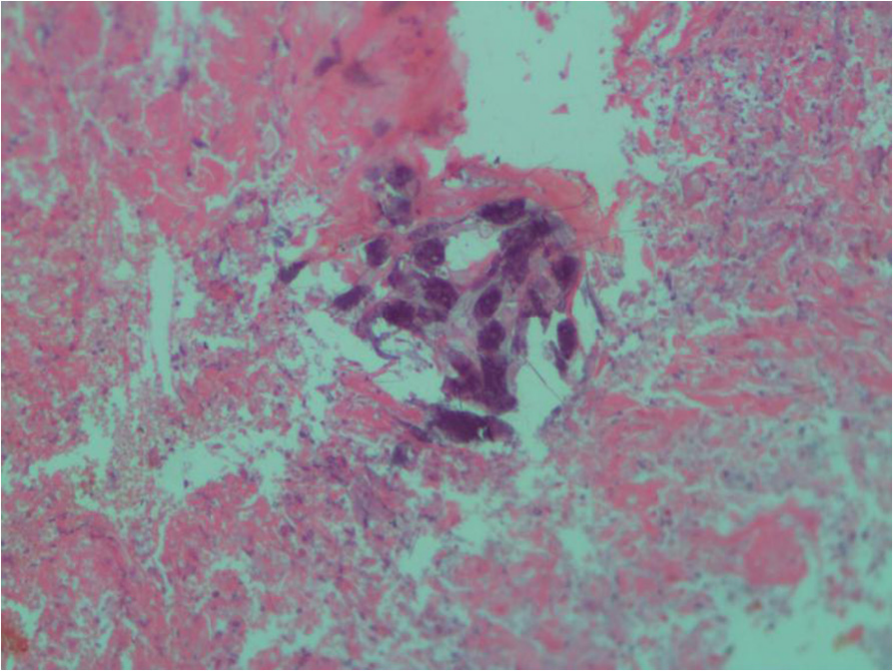
**Arrows showing eggs of *S. haematobium *in the urinary bladder wall of a 40 year old male reported at the Bugando Medical Center, north-western Tanzania with a chief complaint of genital mass and frequent micturitions.** Histological (H &E) examination of urinary bladder biopsy revealed co-infections of urinary bladder cancer and *S. haematobium* eggs in the wall of the bladder and fibrosis.

In pregnancy, *S. mansoni* is not only the cause of hepatosplenomegaly and other morbidities, but has also been associated with anaemia [118]. Heavy *S. mansoni* infection (≥ 400 epg) was associated with anaemia [118]. In hospital reports, *S. mansoni* is the causal factor of appendicitis and intestinal obstructions Figure 4[131].

#### Growth retardation and defects in cognitive function

Schistosomiasis infections during childhood have been associated with growth retardation, anaemia and poor cognitive functions [132-134]. Studies have shown that light to severe infections of either *S. mansoni* or *S. haematobium* are associated with stunted growth and under-nutrition in infected children [132-135]. In Tanzania, heavy infections of either *S. mansoni* or *S. haematobium* were associated with reduced height and weight in infected schoolchildren, in comparison to an uninfected control group [132,133]. Importantly, heavy infection with *S. haematobium* was associated with impaired mental performance or poor cognitive function in Tanzanian school children [134,136]. Evidence from randomized intervention studies indicates that successful chemotherapy results in successful but incomplete catch-up of growth, cognitive function and haemoglobin levels [137].

#### Co-infection of schistosomiasis with other tropical diseases

In Tanzania, co-infection of schistosomiasis with other infectious and parasitic diseases such as malaria, geohelminths and HIV are common [107,110,121,138-141]. In primary school children, double or triple infections of schistosomiasis with either malaria or Soil-Transmitted Helminth infections are common [110,138]. In this age group, malaria and hookworm infections cause and influence schistosomiasis-related morbidities such as hepatosplenomegaly and anaemia [138,142]. Schistosomiasis is reported to influence the severity of malaria infection in school children [143,144]. Co-occurrence of *S. haematobium/S. mansoni* and HIV-1 infections among rural populations has been noted in Tanzania [121], and urogenital schistosomiasis was associated with HIV infection [121]. Emerging putative evidence suggests that there are interactions of schistosomiasis and HIV-1 infections in Africa, with schistosomaisis reported to increase susceptibility to HIV transmission and speed up the progression of HIV/AIDS in Africa [145,146]. The same holds for associations between hepatitis B virus (HBV) and hepatitis C virus (HCV) and *S. mansoni* infection [147]. As noted in previous studies, there is evidence that co-infections of HBV and HCV and *S. mansoni* are associated with deterioration of hepatic function [147].

#### Past and current control of schistosomiasis in Tanzania

From the mid-1950s to 1970s, comprehensive control measures were carried out with an emphasis on the control of the intermediate hosts. Various field trials were conducted in the country, using molluscicides to control intermediate snail hosts of *S. haematobium* and *S. mansoni* species (Table 1) [63,70-74,85,148-151]. The common molluscicides studied and used in control of intermediate snail hosts were N-tritylmorpholine, niclosamide ethanolamine salt, sodium pentachlorophenate and Bayer 73 (2-hydroxy-5, 2-dichloro-4-nitro-benzanilide) [63,70-74,85,148-151]. After several field trials of molluscicides, control programmes started and focused mainly in irrigation schemes and the control approach significantly reduced the incidence of *S. mansoni*[63,70-74,85,148-152]. However, the wide distribution of the intermediate snail host of schistosomiasis and their habitats in the country did not allow large-scale country coverage. In addition, molluscicides were expensive and required substantial human and material resources for efficient application [151]. In addition, the toxic nature of the molluscicides to other aquatic macro and micro-organisms gave rise to ecological concerns.

In the 1960s, the major antischistosomal drugs were antimonials. Various clinical trials were conducted in the country to evaluate their efficacy [153-159]. The main antischistosomes were lucanthone hydrochloride, sodium antimony Dimercaptosuccinate (TWSb) and ambilhar (a nitrothiazole derivative, 1-(5-nitro-2-thiazolyl)-2-imidazole) [153-157]. The parasitological cure rates of the antimonials varied widely. For instance, TWSb for treatment of *S. haematobium* had 90% cure rates among individuals with light infections but in individuals with heavy infection intensity, the cure rate was below 40% [153-155]. In addition to low cure rates, the antimonials were highly toxic, involved prolonged courses of treatment and had very severe side effects such as epileptiform seizures, psychoses, giddiness and epistaxis [153-157]. Thus, most of these drugs were not recommended for community mass treatment.

The current global strategy of schistosomiasis control is to reduce morbidity by decreasing worm burden and intensity of infection. The strategies aim to improve children’s health and prevent irreversible complications in adulthood. This has become possible by the use of praziquantel, and the national control programme uses it [160]. In 1986, a control programme for urogenital schistosomiasis was initiated on the island of Pemba (Pemba Schistosomiasis Control Programme, 1986–1990) [23-27]. In 2003 the new Schistosomiasis Control Programme (“Kick out Kichocho”) was launched in Unguja. The two programs targeted school children and offered praziquantel treatment. The report of Schistosomiasis Control Initiatives indicates that for the period of three years (2004–2006), Zanzibar had successfully completed a countrywide coverage treatment of all school children, using praziquantel (http://www.sci-ntds.org). At present, SCORE is supporting an ambitious operation research in Pemba and Unguja aiming at (i) eliminating schistosomiasis in Unguja in the coming three years and interrupting transmission in the next five years (ii) to control schistosomiasis in Pemba (prevalence < 10%) in the next three years and to eliminate the disease in the next five years. The project was implemented in 2011 under the within the context of ongoing national control and prevention efforts. Zanzibar Elimination of Schistosomiasis Transmission (ZEST) is working to achieve these common goals and their results are highly waited. A recent report from Unguja have indicated that the prevalence of *S. haematobium* and other helminth infections have markedly declined on the island for the past 20–25 years and this was attributed to the morbidity control programmes [161]. These findings are encouraging and call for the need of integrating control approach to meet the objectives of the SCORE project.

In 2004, the National Schistosomiasis and Soil-transmitted Helminths Control Programme (NSSCP) under the Ministry of Health and Social Welfare was established with support from the Schistosomiasis Control Initiative (SCI) on the mainland part of Tanzania [162,163]. According to an NSSCP report, the school-based Mass Deworming Treatment (MDA) has covered 21 regions and each region has received one, two or three rounds of treatment [162].

In the country, various approaches have been used for delivering treatment to endemic communities. The main approach is the school-based MDA, where all primary school children receive praziquantel (40 mg/kgBWT) irrespective of infection status (blanket mass treatment) [162]. In this approach, school teachers administer treatment to the school age children in their respective schools. The school-based deworming programme is advocated as a highly cost effective public health intervention [162]. The other approach is the community-directed treatment (ComDT) approach, where communities are empowered to decide their own methods of distributing drugs, at their convenient time and ideal places of delivery [111,164]. In reported cases, the communities selected people whom they believe would be able to distribute drugs to the children after training [164]. The ComDT approach was at least as effective as the school-based approach in reducing prevalence and intensity of schistosomiasis among school children [164].

Public health education focusing on behavioral change, treatment-seeking and sanitation forms an important part of control programmes for schistosomiasis, especially in rural villages. Knowledge of local perceptions and practices are important in designing and implementation of any of the schistosomiasis control approaches [165]. At community level, qualitative and quantitative studies have reported a fairly good knowledge of symptoms and health-seeking behaviour for schistosomiasis [165,166]. On the other hand, poor knowledge on the causes of schistosomiasis was observed [165,166]. Various methods of health education have been tried and practiced in risk groups such as school children, for instance, in Unguja, to increase awareness and knowledge of the school children about schistosomiasis. A booklet titled “*Juma na kichocho*” (Juma and schistosomiasis) was developed as a teaching aid [167]. However, the information from the booklet did not provide significant changes in knowledge and attitudes of the school children [167]. This was similar to other observations around the Lake Victoria area, where the posters about schistosomiasis provided in primary schools as a source of information did not influence the children’s knowledge about the disease [166].

#### Future control of schistosomiasis: Integrated control approach

Considering the wide distribution of schistosomiasis in the country and the wide media of transmission, an integrated and sustainable approach is required to control the disease and its associated morbidities. A combination of approaches could be used in human hosts and the intermediate hosts.

For human hosts, repeated mass chemotherapy using praziquantel, public health education focusing on behaviour changes towards risk factors, improve sanitation/hygiene-constructions and use of toilets and supplying tap water in risk groups such as fishing and farming communities, changes of agriculture practices – cultivation in flooded paddy fields and sugarcane plantations and use of gumboots are important measures.

For the intermediate hosts, taking into consideration the seasonal fluctuation of population density of intermediates hosts, timed and selective mollusciciding before aestivation begins, and treatment of snail’s habitats at the beginning of the rainy seasons would help to control the disease in small scale [16]. Furthermore, biological control- use of insects or fish, which will feed on snails, or use of competitor snails, is another option for small scale control [72,73].

Recently, integrated control approaches, which used praziquantel treatment along with environmental improvement, water and sanitation, snail control and public health education activities have been reported to be highly effective, and the prevalence of schistosomiasis japonicum infection in human declined to <1% in China and in regions of Indonesia (from 4.66% and 4.8% to 0.89% and 0.3% respectively) [160,168].

The WHO, through its fifty-fourth World Health Assembly resolution, has endorsed chemotherapy with praziquantel as a public health strategy to control schistosomiasis related morbidities [160]. In addition, the resolution has recognized the importance of other public health measures such as the use of safe tape water, improving sanitation and public health education as means to control schistosomiasis [160].

## Conclusions

In general, Tanzania has made collective steps to understand the transmission dynamics and control efforts of schistosomiasis over the past seven decades. The evidence revealed here indicates that despite considerable efforts made to control schistosomiasis using various approaches in Tanzania, the disease remains a serious public health problem and its prevalence continues to increase with increase in the population size.

Currently, to respond to the sixty-fifth World Health Assembly resolution, which called for endemic countries and the international community to allocate resources for intensification of schistosomaisis control activities [160], there are new efforts to control schistosomiasis in Tanzania. The funding provided by the Bill and Melinda Gates Foundation and to the Schistosomiasis Initiatives Control (SCI), and the commitment of the Tanzanian government through the Ministry of Health and Social Welfare, has enabled millions of Tanzanian school children to receive at least one to three rounds of praziquantel treatment [162]. In addition, the call of the central government of Tanzania to local district authorities to allocate budget for controlling schistosomiasis, especially for buying and distributing drugs (praziquantel) to risk groups (primary school children) are encouraging measures [169]. However, a single approach of preventive chemotherapy using praziquantel alone cannot overcome the problem of schistosomiasis in Tanzania. There is a need for integrated control programs, acting beyond preventive chemotherapy [160]. In fact, there is a need for deliberate efforts to enforce health education, and more action is needed in improving access and supply of clean tap water and adequate sanitation (especially the use of toilets) [160].

For preventive chemotherapy, there is a need to expand treatment programs to other risk groups such as fishermen, farmers and non-school going children. Furthermore, with the expansion of praziquantel distribution under SCI support (from 2004–2007, about 36.75 million praziquantel tablets were received in Tanzania), there is a need to understand the best approach to distribute the drugs to communities living in various endemic levels of schistosomaisis. Another SCORE funded project is also going on around the Lake Victoria aiming at understand the best approach to distribute anthelminthic to communities with different endemicity level. We hope that the two control projects will yield fruitful results for the future control of the disease.

Lastly, in view of the fact that schistosomiasis occurs in rural areas where the majority of the population are highly stricken by poverty, improvement of the life standard of these communities should go hand in hand with schistosomaisis control activities.

## Competing interests

HDM is supported by the Training Health Researchers into Vocational Excellence in East Africa(THRiVE) Programme funded by Wellcome Trust, grant number 087540. The authors declare no conflicts of interest.

## Authors’ contributions

HDM designed the review, conducted the literature search and drafted the first version of the manuscript. All authors read and approved the manuscript and contributed to its content.
